# Monte Carlo studies on photon interactions in radiobiological experiments

**DOI:** 10.1371/journal.pone.0193575

**Published:** 2018-03-21

**Authors:** Mehrdad Shahmohammadi Beni, D. Krstic, D. Nikezic, K. N. Yu

**Affiliations:** 1 Department of Physics, City University of Hong Kong, Tat Chee Avenue, Kowloon Tong, Hong Kong; 2 Faculty of Science, University of Kragujevac, Kragujevac, Serbia; Ludwig-Maximilians-Universitat Munchen, GERMANY

## Abstract

X-ray and γ-ray photons have been widely used for studying radiobiological effects of ionizing radiations. Photons are indirectly ionizing radiations so they need to set in motion electrons (which are a directly ionizing radiation) to perform the ionizations. When the photon dose decreases to below a certain limit, the number of electrons set in motion will become so small that not all cells in an “exposed” cell population can get at least one electron hit. When some cells in a cell population are not hit by a directly ionizing radiation (in other words not irradiated), there will be rescue effect between the irradiated cells and non-irradiated cells, and the resultant radiobiological effect observed for the “exposed” cell population will be different. In the present paper, the mechanisms underlying photon interactions in radiobiological experiments were studied using our developed NRUphoton computer code, which was benchmarked against the MCNP5 code by comparing the photon dose delivered to the cell layer underneath the water medium. The following conclusions were reached: (1) The interaction fractions decreased in the following order: ^16^O > ^12^C > ^14^N > ^1^H. Bulges in the interaction fractions (versus water medium thickness) were observed, which reflected changes in the energies of the propagating photons due to traversals of different amount of water medium as well as changes in the energy-dependent photon interaction cross-sections. (2) Photoelectric interaction and incoherent scattering dominated for lower-energy (10 keV) and high-energy (100 keV and 1 MeV) incident photons. (3) The fractions of electron ejection from different nuclei were mainly governed by the photoelectric effect cross-sections, and the fractions from the 1s subshell were the largest. (4) The penetration fractions in general decreased with increasing medium thickness, and increased with increasing incident photon energy, the latter being explained by the corresponding reduction in interaction cross-sections. (5) The areas under the angular distribution curves of photons exiting the medium layer and subsequently undergoing interactions within the cell layer became smaller for larger incident photon energies. (6) The number of cells suffering at least one electron hit increased with the administered dose. For larger incident photon energies, the numbers of cells suffering at least one electron hit became smaller, which was attributed to the reduction in the photon interaction cross-section. These results highlighted the importance of the administered dose in radiobiological experiments. In particular, the threshold administered doses at which all cells in the exposed cell array suffered at least one electron hit might provide hints on explaining the intriguing observation that radiation-induced cancers can be statistically detected only above the threshold value of ~100 mSv, and thus on reconciling controversies over the linear no-threshold model.

## Introduction

When alpha-particles or heavy ions (which are directly ionizing radiations) are used to study radiobiological effects of ionizing radiations, researchers are cautious about the percentage of cells in the cell population which are actually hit by the particles. In the present paper, a demarcation between “exposed” cells and “irradiated” cells was necessary. Irradiated cells referred to the cells which had been hit by at least one directly ionizing radiation (such as alpha-particles, heavy ions or electrons) while non-irradiated cells referred to those which had not been hit by any directly ionizing radiation. As such, when a cell population was exposed, it was possible that some cells were irradiated while some cells remained unirradiated. The percentage of irradiated cells in an exposed cell population is particularly important with the discovery of the “rescue effect”. The rescue effect described the phenomenon that irradiated cells derived benefits from feedback signals released from bystander unirradiated cells, e.g., the unirradiated cells could alleviate the harmful radiobiological effects in the irradiated cells. In 2011, the rescue effect between human primary fibroblast and human cervical cancer (HeLa) cells was revealed [1]. The rescue effect was subsequently demonstrated in different *in vitro* [2–8] and *in vivo* [9,10] experiments. Some studies also demonstrated a deviant rescue effect which led to exacerbated harmful radiobiological effects in the irradiated cells [11,12]. Studies on the rescue effect and possible mechanisms messengers were reviewed [13]. In particular, rescue effect was found to be induced in α-particle-irradiated HeLa and NIH/3T3 cells through activation of the nuclear factor kappa B (NF-κB) pathway in the irradiated cells [5].

X-ray and γ-ray photons have also been widely used for studying radiobiological effects of ionizing radiations. It is well established that photons are indirectly ionizing radiations, which means that they need to set in motion electrons (a directly ionizing radiation) to perform the ionizations. Apparently, researchers are less cautious about the percentage of cells in the cell population which are actually hit by these electrons, probably because of the seemingly “pervasive” nature of photons. Nevertheless, it is expected that when the photon dose decreases to below a certain limit, the number of electrons set in motion will become so small that not all cells in the “exposed” cell population will get at least one electron hit. When some cells in the cell population are not irradiated, there can be rescue effect between the irradiated cells and non-irradiated cells, and the resultant radiobiological effect observed for the “exposed” cell population will be different. It is therefore pertinent to study in more detail the characteristics of photon interactions in radiobiological experiments, in particular these dose limits which also depend on the energy of the photons. The present paper is devoted to these studies through Monte Carlo studies.

## Monte Carlo method and NRUphoton code

**Fig 1:**
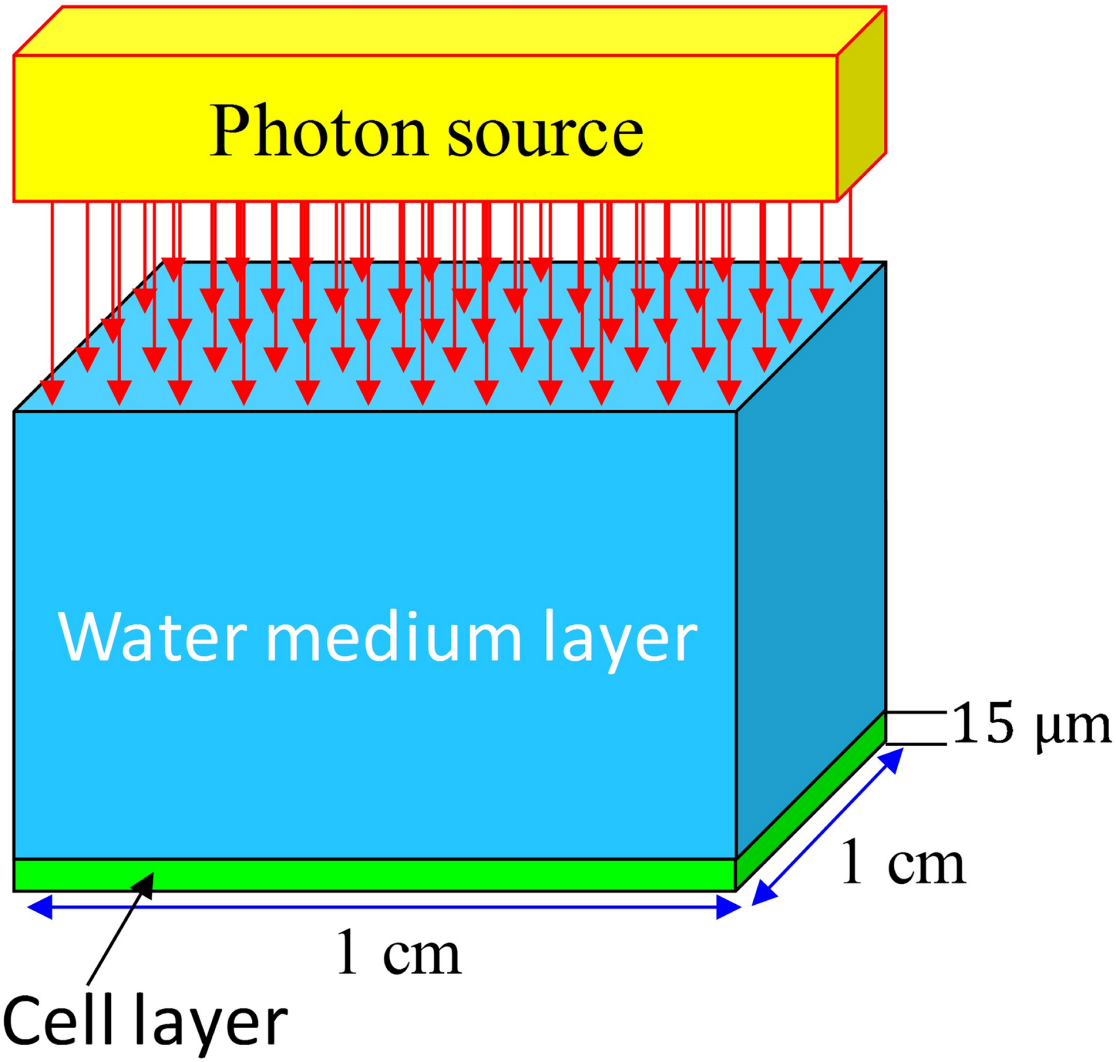
Schematic diagram showing the photon irradiation setup.

The experimental setup is schematically illustrated in Fig 1. Photon propagation from the medium layer to the cell layer was simulated using the Monte Carlo method. Subsequent photon interactions with different nuclei in both layers were also simulated.

**Fig 2:**
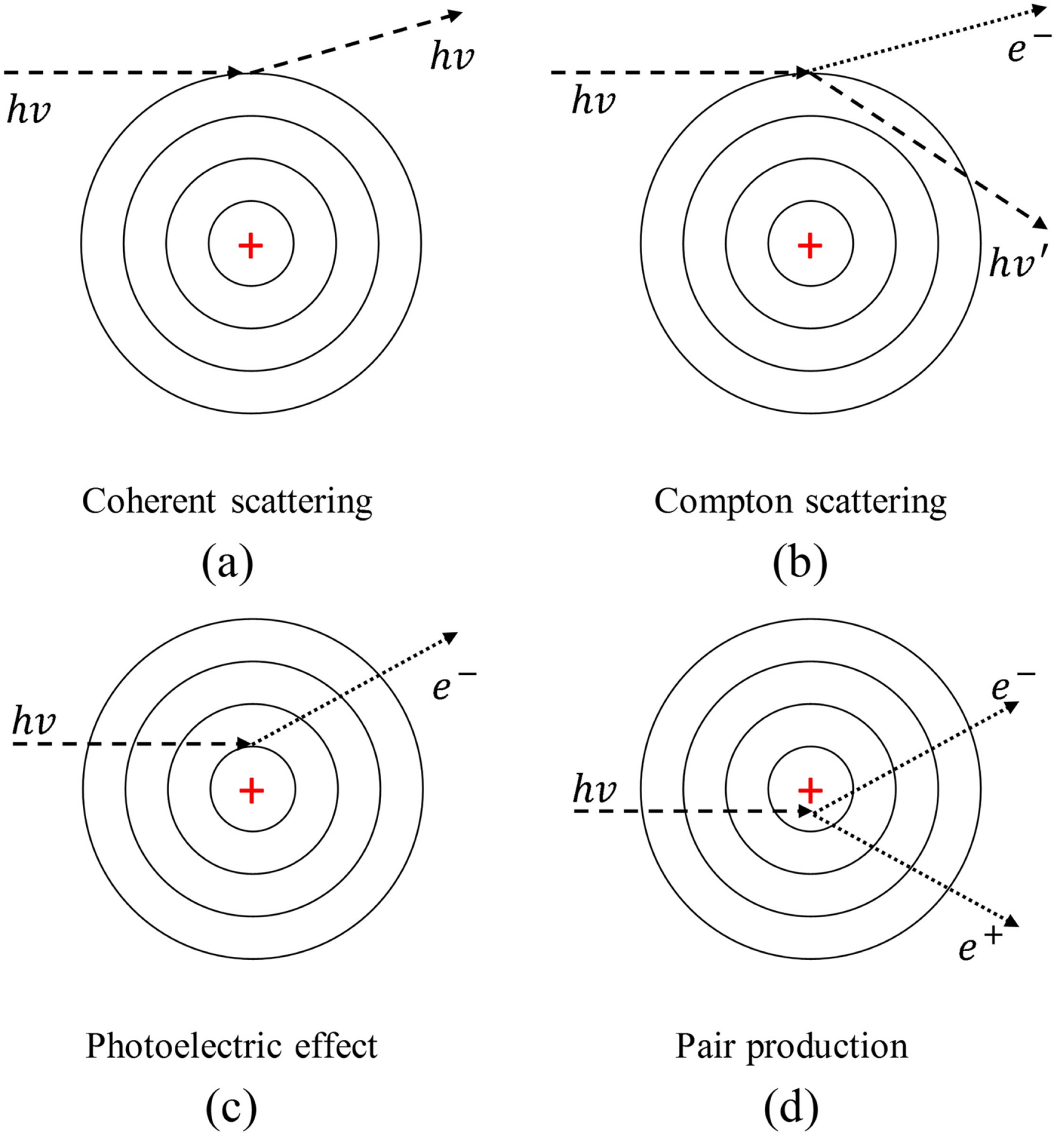
Schematic diagrams of photon interaction mechanisms considered in the NRUphoton code.

The NRUphoton code was designed for simulation of photon interactions and was modified from our previous NRUneutron computer program which was prepared using Fortran90. The present version of the NRUphoton code consisted of a pure photon transport mode and local energy deposition at the photon interaction sites was assumed. The photon interaction mechanisms considered in the NRUphoton code were (1) coherent scattering, (2) incoherent scattering, (3) photoelectric effect and (4) pair production, which are schematically shown in Fig 2.

The present computer program was capable of simulating photon energies up to 10 MeV with a lower photon energy cutoff of 1 keV. The inputs to the NRUphoton code included (1) photon energy, (2) total number of photons launched (NPS), (3) incident angles and (4) dimensions of the medium-layer and cell-layer domains (*x*, *y*, *z*). The energy dependent photo-atomic cross-section data were also required. Water was made up of ^1^H and ^16^O, while the cells were emulated by a tissue equivalent plastic (density = 1.127 g/cm^3^) made up of ^12^C, ^1^H, ^14^N and ^16^O (with mass percentages of 11.1, 10.1, 2.6 and 76.2, respectively). Depending on realistic situations, other compositions, e.g., those from Ref [14], could also be employed.

### Energy dependent photon cross-section data

**Fig 3:**
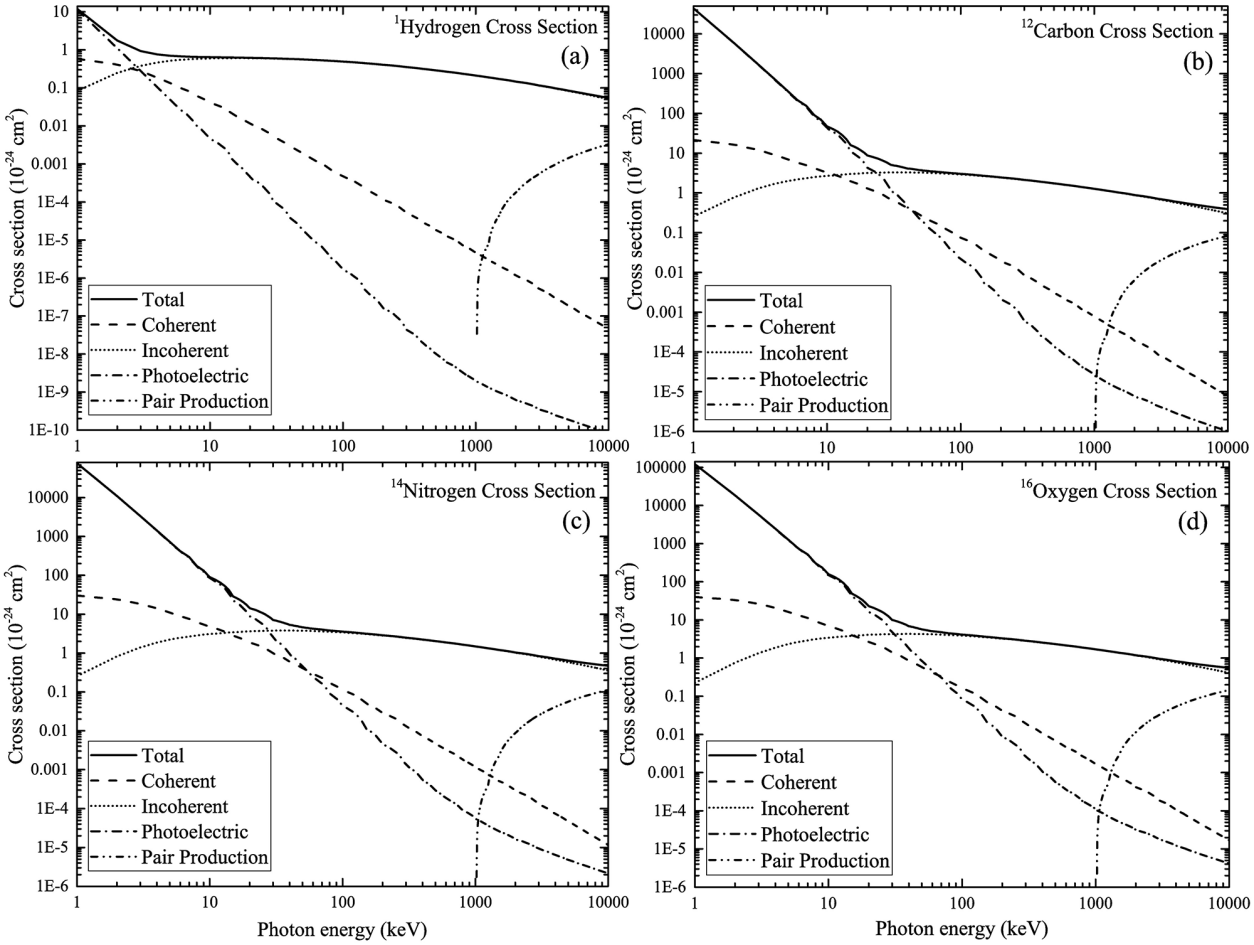
Energy-dependent photo-atomic cross-section data for (a) ^1^H, (b) ^12^C, (c) ^14^N and (d) ^16^O nuclei.

The total energy-dependent photo-atomic cross-section data and energy-dependent cross-sections for each interaction mechanism shown in Fig 2 were obtained from the National Institute of Standards and Technology (NIST) XCOM library homepage: https://physics.nist.gov/PhysRefData/Xcom/html/xcom1.html. Similar to our previous works [15,16], photons with energies between 1 keV and 10 MeV were considered. The total photon cross-sections for ^1^H, ^12^C, ^14^N and ^16^O nuclei were obtained from the XCOM library homepage. The percentage of each mechanism for the ionizing photon energies of 10 keV, 100 keV and 1 MeV are summarized in Table 1. Linear interpolation of cross-section data was needed. The cross-section data used in the NRUphoton code are shown in Fig 3. The total macroscopic photon interaction cross sections for water and cell layers were calculated using these data.

**Table 1:** Percentage of photon interaction mechanisms (coherent scattering, incoherent scattering and photoelectric effect) for the ionizing photon energies of 10 keV, 100 keV and 1 MeV, obtained from photo-atomic cross-section data.

Nuclei	Incident photon Energy	Coherent (%)	Incoherent (%)	Photoelectric (%)
^1^H	10 keV	6.398	92.90	0.7069
	100 keV	0.09373	99.91	0.0003700
	1 MeV	0.02190	99.99	0.00001000
^12^C	10 keV	6.829	5.698	87.47
	100 keV	2.457	96.86	0.6814
	1 MeV	0.06061	99.94	0.002090
^14^N	10 keV	5.234	3.428	91.34
	100 keV	3.118	95.66	1.222
	1 MeV	0.07838	99.92	0.003820
^16^O	10 keV	4.307	2.174	93.52
	100 keV	3.860	94.14	2.005
	1 MeV	0.09864	99.89	0.006460

### Geometry and photon tracking module

Similar to our previous work, the cross-sectional area of water and cell layers was fixed at 1×1 cm^2^ and the cell thickness was chosen to be 15 μm. The variation in the characteristics of photons interacting with the radiobiological system (Fig 1) were investigated for ten water layer thicknesses between 500 and 5000 μm [17]. Here, the primary interaction site of photons with initial energy of *E*_*0*_ in the water medium was denoted as (*x*_*0*_, *y*_*0*_, *z*_*0*_). The mean free path of the launched photons was first calculated, which determined the domain in which the photon interaction took place. After that, the nuclei involved in the photon interaction were determined based on the energy dependent cross-section data. The interaction mechanism was then chosen through calling the “INTERTYPE” subroutine which was developed and bundled with the NRUphoton code. Inputs to the INTERTYPE subroutine included (1) photon energy, (2) cross-section data for specific interaction mechanism (see Fig 2), (3) atomic numbers of the nuclei and (4) binding energies of the main atomic subshells for the nuclei involved in the photon interactions. The INTERTYPE subroutine determined the energy and the scattering angle of the scattered photons.

In the coherent scattering event, the energy of the photon remained unaltered but its direction would change. The angular distribution of photons upon coherent scattering was described by the product of the atomic form factors, *F(q*,*z)* with the classical Thompson cross-section

$$\frac{d\sigma }{d\Omega }=\frac{r_{e}^{2}}{2}(1+{\text{cos}}^{2}\theta )$$ (1)

where *r*_*e*_ was the classical electron radius and *θ* was the scattering angle. The relativistic Hartree-Fock atomic form factors were incorporated into the coherent scattering cross-section data, which would account for the correction of the Thompson scattering angle distribution. The cumulative distribution function (CDF) was obtained as

$$CDF_{coherent}=\frac{1}{2}-\frac{1}{8}{\text{cos}}^{3}\theta -\frac{3}{8}\text{cos}\theta$$ (2)

from which the angular distribution of photons undergoing coherent scattering were sampled.

In the incoherent scattering event, both energy and direction of the photon changed, and was described by the Klein-Nishina theory. In the Klein-Nishina formulation, the electron was assumed to be initially free and at rest [18], so the Klein-Nishina differential microscopic cross-section was

$$\frac{d\sigma^{*}(\alpha_{0},\theta )}{d\Omega }=\frac{1}{2}r_{e}^{2}{\left[1+\alpha_{0}(1-\text{cos}\theta )\right]}^{-2}\times [1+{\text{cos}}^{2}\theta +\frac{\alpha_{0}^{2}{\left(1-\text{cos}\theta \right)}^{2}}{1+\alpha_{0}(1-\text{cos}\theta )}]$$ (3)

where *α*_*0*_ was the energy of the incoming photon expressed in the unit of electron rest mass, *r*_*e*_ was the classical electron radius and *θ* was the scattering angle. The scattering angle and the energy of the photon after an incoherent scattering were sampled using the Carlson method [18], where an accurate fit to the inverse CDF was developed, which could be used for photon energies ≤ 2 MeV. The energy of the photon after incoherent scattering was

$$\alpha =\frac{\alpha_{0}}{1+(\frac{\alpha_{0}}{1+0.5625\alpha_{0}})\gamma +(2\alpha_{0}-(\frac{\alpha_{0}}{1+0.5625\alpha_{0}}))\gamma^{3}}$$ (4)

where *α*_*0*_ was the incoming photon energy expressed in the unit of electron rest mass and *γ* was a uniformly distributed random number between 0 and 1. Upon computation of the photon energy after incoherent scattering, the scattering angle of the photon was obtained as

$$\frac{\alpha }{\alpha_{0}}=\frac{1}{1+\alpha_{0}(1-\text{cos}\theta )}$$ (5)

In the photoelectric interaction, the concerned photon disappeared and the tracking module for that photon was terminated. The energy transferred to an atomic electron was given by the difference between the energy of the incoming photon and the electron binding energy of the specific atomic subshell. The atomic subshell photoionization cross-section was calculated from the Hartree-Fock-Slater one-electron central potential model, i.e., the dipole approximation [19–21]:

$$\sigma_{nl}(E)=\frac{4\pi^{2}\alpha_{0}a_{0}^{2}}{3}\frac{N_{nl}}{2l+1}E[lR_{l-1}^{2}(E_{kin})+(l+1)R_{l+1}^{2}(E_{kin})]$$ (6)

where *α*_*0*_ was the fine structure constant, *a*_*0*_ was the Bohr radius, *N*_*nl*_ was the number of electrons in the *nl* subshell, *R*_*l±1*_*(E*_*kin*_*)* were one-electron radial dipole matrix elements; these were assumed to be between the initial discrete and final continuum energy states of *ϕ*_*nl*_ and *ϕ*_*ϵ*,*l*’_, respectively. The parameter *E*_*kin*_ was the kinetic energy of the ionized electron (i.e., difference between the incoming photon energy and the electron binding energy in the *nl*^*th*^ subshell). Considering the dipole length approximation, the matrix element *R*_*l±1*_ could be expressed as

$$R_{l\pm 1}=\int_{0}^{\infty }P_{nl}\left(r\right)rP_{\epsilon ,l\pm 1}\left(r\right)dr$$ (7)

where *P*_*nl*_*(r)/r* and *P*_*ϵ*,*l±1*_*(r)/r* were the initial and final single-particle wave functions. The *R*_*l±1*_ matrix elements in dipole velocity and acceleration approximation were

$$R_{l\pm 1}=\frac{2}{\epsilon -E_{nl}}\int_{0}^{\infty }P_{nl}\left(r\right)\left[\frac{d}{dr}\pm \frac{2l+1\pm 1}{2r}\right]P_{\epsilon ,l\pm 1}\left(r\right)dr$$ (8)

$$R_{l\pm 1}=\frac{4}{{\left(\epsilon -E_{nl}\right)}^{2}}\int_{0}^{\infty }P_{nl}\left(r\right)\frac{dV_{nl}\left(r\right)}{dr}P_{\epsilon ,l\pm 1}\left(r\right)dr$$ (9)

where *E*_*nl*_ was the binding energy of the electron in the *nl*^*th*^ subshell. Considering the main assumption in the Hartree-Fock-Slater approximation which was: “*the initial state of an electron is in a central field potential*”, the one-electron Schrödinger equation could be abridged as

$$(\frac{d^{2}}{dr^{2}}+V(r)+E_{nl}-\frac{l(l+1)}{r^{2}})P_{nl}(r)=0$$ (10)

where *V(r)* was the sum of Columbic and free electron exchange potential. The final state could be approximated with the same potential and the only difference was that the electron was removed to a continuum state with *E*_*kin*_ > 0. The Schrödinger equation could thus be written as

$$(\frac{d^{2}}{dr^{2}}+V(r)+E_{kin}-\frac{l^{\prime }(l^{\prime }+1)}{r^{2}})P_{\epsilon ,l^{\prime }}(r)=0$$ (11)

where *V(r)* was the same potential as that in Eq (10) and *l*^*’*^ = *l* ±*1* by considering the conservation of angular momentum. Upon the photoelectric interaction, our present code would determine the atomic subshell at which the electron was ejected. The atomic subshells considered were 1s(1)H, 1s(1)C, 2s(2)C, 2p(2)C, 1s(1)N, 2s(2)N, 2p(3)N, 1s(2)O, 2s(2)O and 2p(4)O [22].

In the pair production event, the photon disappeared so the tracking and transport modules would be terminated. The threshold energy of the photon required to create the electron-positron pair was *2m*_*e*_*c*^*2*^ (i.e., sum of the rest mass energy of the created particles). The cross-section for pair production increased in a monotonic manner for photon energies above the threshold. The generated positron (*e*^+^) would annihilate with an electron to emit two photons. Since the mean free path of the generated positron was negligible compared to that for photons, the tracking and transport of the generated photons as a result of annihilation could be started at the site at which pair production initially occurred. In the present version of the NRUphoton code, the location and number of pair production events could be scored. However, due to the complexities involved in the pair production events such as tracking of annihilation photons, the maximum photon energy was limited to be 1 MeV so pair production would not occur.

Event-by-event three-dimensional tracking of propagation of particles in water and cell domains were described in detail in our previous works [15,16], and would not be repeated here.

## Benchmarking of NRUphoton code

**Fig 4:**
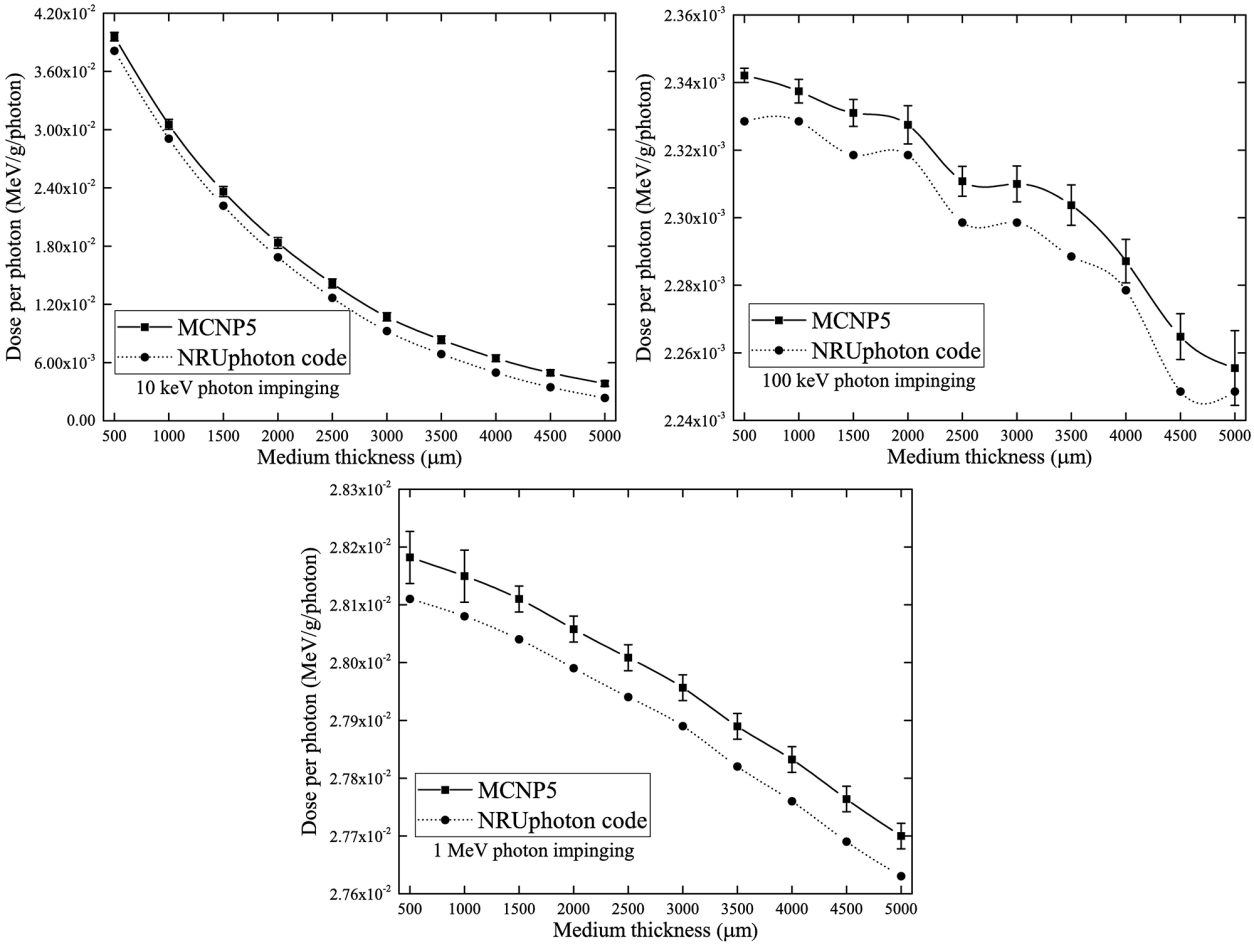
Absorbed photon doses in the cell layer for three incident photon energies of 10 keV, 100 keV and 1 MeV impinging perpendicularly onto the water medium layer with varying thickness computed using the MCNP5 and NRUphoton code.

Similar to our previous work, the computer code developed in the present work was benchmarked against the Monte Carlo N-Particle (MCNP) 5 code [23]. Both computer codes utilized pointwise cross-section data. The photon dose absorbed in the cell layer underneath the water medium layer was used to verify the accuracy and reliability of the present code. The photon dose was calculated for all interactions which took place along the photon trajectory and local energy deposition was assumed for all photon interactions. In other words, the created secondary electrons were not transported away from their creation site. The results of this benchmarking for the three incident photon energies of 10 keV, 100 keV and 1 MeV are shown in Fig 4(a) to 4(c). The results showed good agreement between the MCNP5 and NRUphoton codes. Deviations between the results obtained using the two codes were mainly due to the different cross-section data employed, namely, ENDF/B-VI.8 data library used in MCNP5. The different photon cross-section libraries show considerable variations for different photon energies and interaction mechanisms [24], so the simulation results could be different through selecting different data. Interested readers are referred to Ref. [24] and references therein for comparisons between different photon cross-section libraries. The XCOM offered a user-friendly online platform for users to download the cross-section files in the ASCII format using their provided energy grid. From the NIST website (https://physics.nist.gov/PhysRefData/Xcom/html/xcom1.html), it was also possible to obtain the cross-section data for a compound/mixture. The smaller photon dose absorbed in the cell layer for a thicker water medium was explained by the reduction in photon energy that interacted with the cell layer. A similar trend was also obtained in our previous work [25].

## Computation scheme and outputs

In the present work, the thickness of cells was fixed at 15 μm. The effect of water medium thickness for three photon energies (namely, 10 keV, 100 keV and 1 MeV) on the following photon interaction characteristics were thoroughly investigated:

1. Interaction fraction of photons with ^1^H, ^12^C, ^14^N and ^16^O in the cell layer;
2. Fraction of interaction mechanisms of photons;
3. Fraction of electron ejection from different atomic subshells during photoelectric interaction;
4. Penetration fraction of photons;
5. Angular distribution of photons exiting the water layer and subsequently undergoing interactions within the cell layer;
6. Number of electron hits within a cell array and its dependence on administered dose.

### Interaction fraction of photons with various nuclei within cell layer

**Fig 5:**
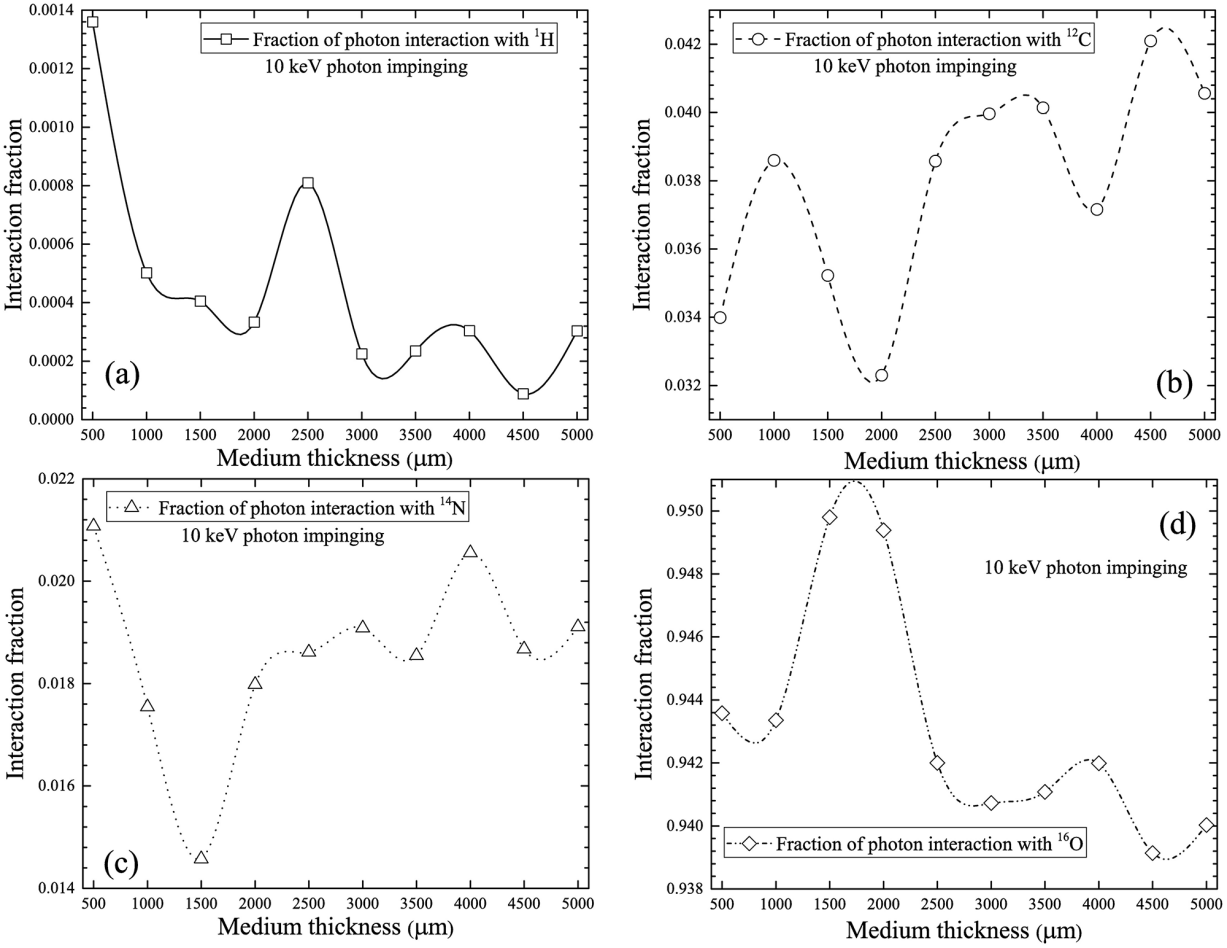
Interaction fractions of photons with (a) ^1^H, (b) ^12^C, (c) ^14^N and (d) ^16^O within cell layer for 10 keV incident photons.

The normalized number of photon interactions with ^1^H, ^12^C, ^14^N and ^16^O were computed. The results obtained for photons with incident energy of 10 keV are presented in Fig 5. The interaction fraction of 10 keV incident photons with ^16^O nuclei was the highest compared to other nuclei mainly due to the significantly larger ^16^O nuclei cross-section (see Fig 3d) and also to its abundant presence in the currently considered cell composition. The interaction fraction with ^12^C nuclei was the second highest, which was explained by its relatively higher cross-section and also by the adopted cell composition. The third highest interaction fraction occurred for the ^14^N nuclei. Despite the similar cross-section of ^14^N compared to ^12^C, the present cell composition consisted of only 2.6% ^14^N. The small interaction cross-section of ^1^H nuclei accounted for its smallest interaction fraction (see Fig 3a). In Fig 5, bulges in the interaction fractions were observed, which reflected changes in energies of the propagating photons as a result of the water medium thickness as well as changes in the energy-dependent photon interaction cross-sections. In general, the numbers of photon interactions with different nuclei in the cell layer were governed by their respective interaction cross-sections and their abundance in the layer. The interaction fractions decreased in the following order: ^16^O > ^12^C > ^14^N > ^1^H.

**Fig 6:**
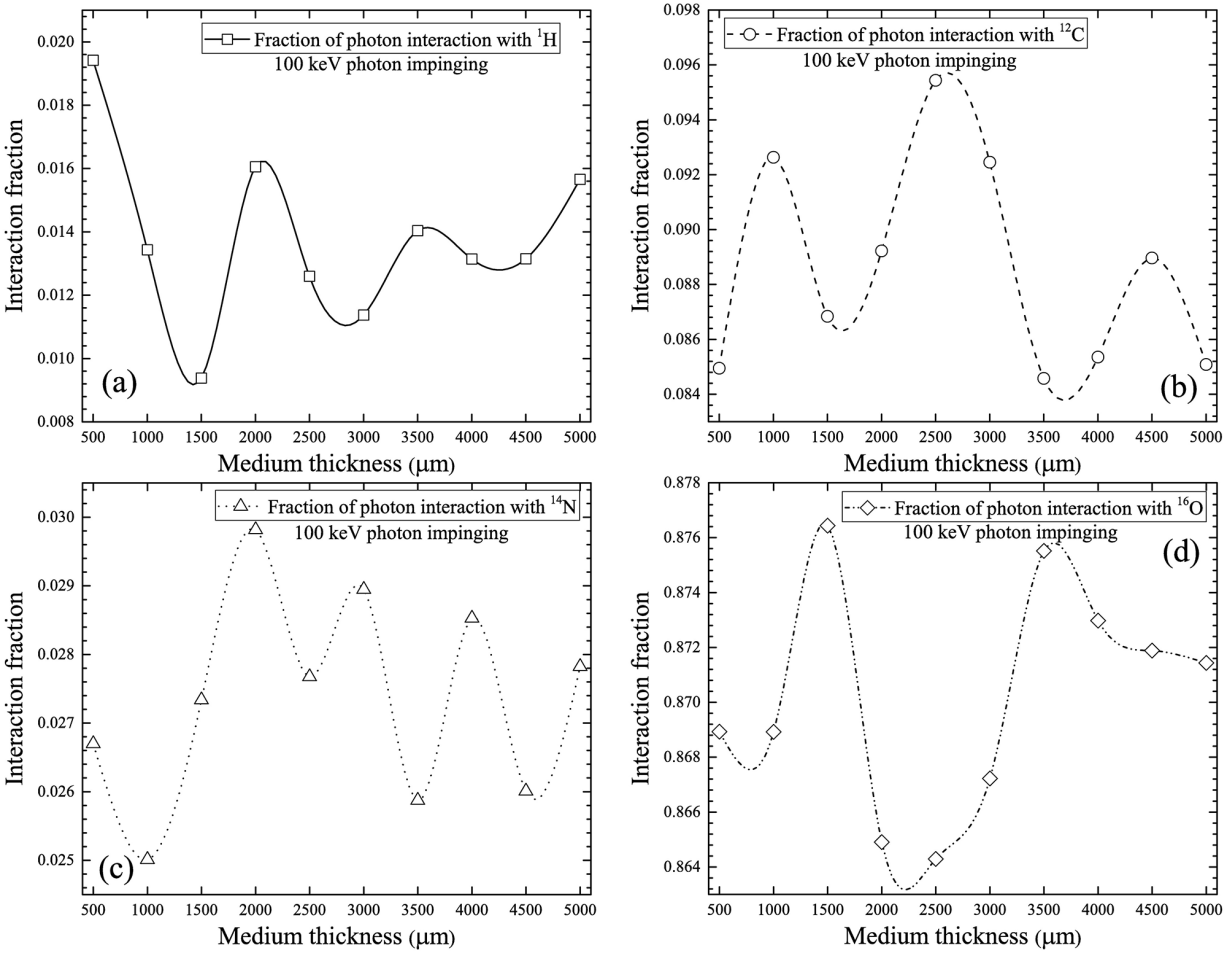
Interaction fractions of photons with (a) ^1^H, (b) ^12^C, (c) ^14^N and (d) ^16^O within cell layer for 100 keV incident photons.

The interaction fractions for different nuclei in the cell layer for 100 keV incident photons are shown in Fig 6. The trends were similar to those for 10 keV incident photons. The order of decrease: ^16^O > ^12^C > ^14^N > ^1^H remained the same. However, the magnitudes of the interaction fractions were different from those for 10 keV photons, which was due to different interaction cross-sections. The bulges shown in Fig 6 were more conspicuous than those shown in Fig 5. At higher photon energies, incoherent scattering was more efficient, when compared to lower photon energies at which photoelectric effect dominated, which did not cause scattering.

**Fig 7:**
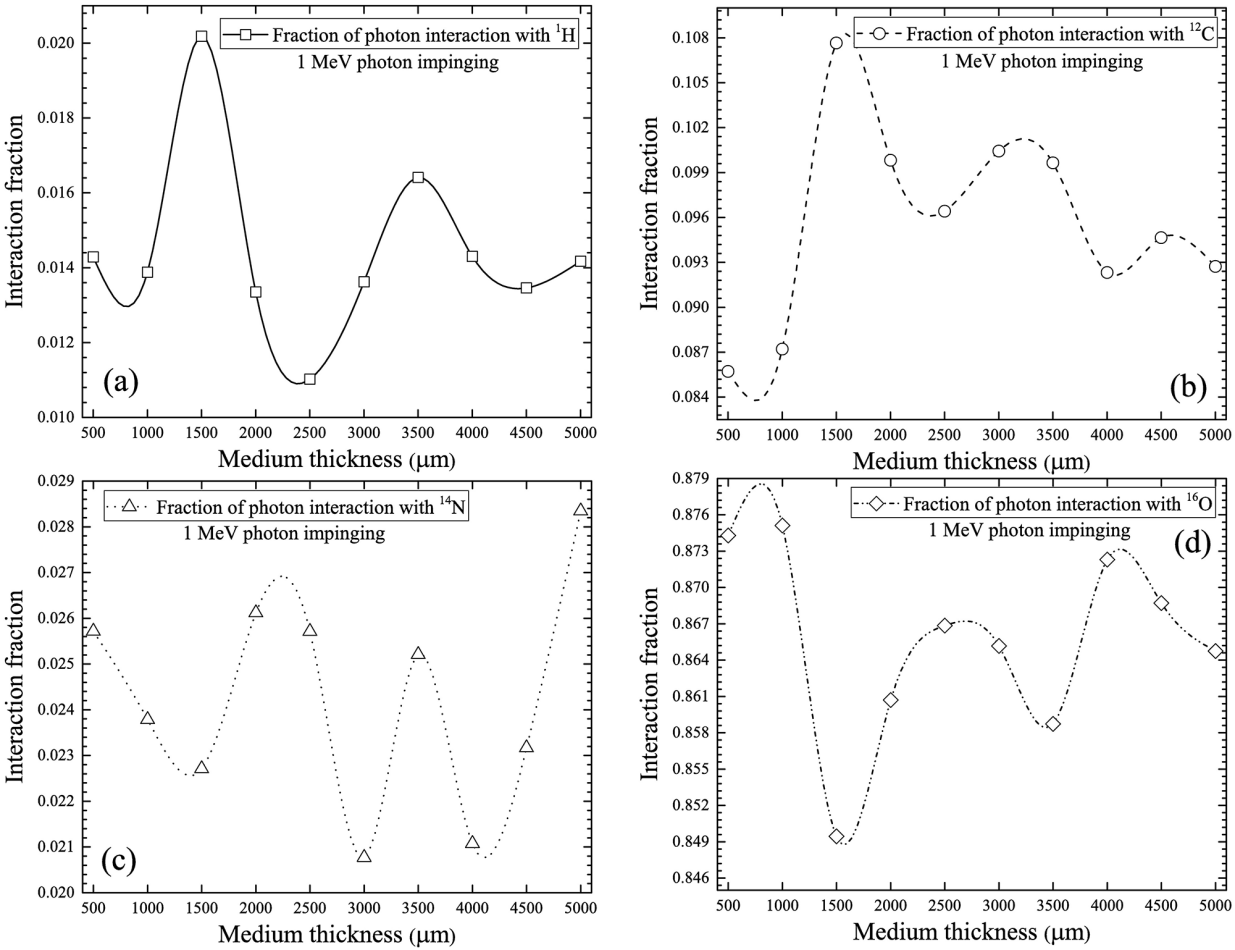
Interaction fractions of photons with (a) ^1^H, (b) ^12^C, (c) ^14^N and (d) ^16^O within cell layer for 1 MeV incident photons.

The interaction fractions for different nuclei in the cell layer for 1 MeV incident photons are shown in Fig 7. The order of decrease: ^16^O > ^12^C > ^14^N > ^1^H remained the same. For photons with this incident energy, the main interaction mechanism of photons was incoherent scattering (see Fig 3). It was interesting to note the similarity between the trend and the magnitudes of interaction fractions for 100 keV and 1 MeV photons (see Figs 6 and 7), which was attributed to the same dominating interaction mechanism (incoherent scattering) for these two photon energies.

### Fractions of interaction mechanisms of photons

**Fig 8:**
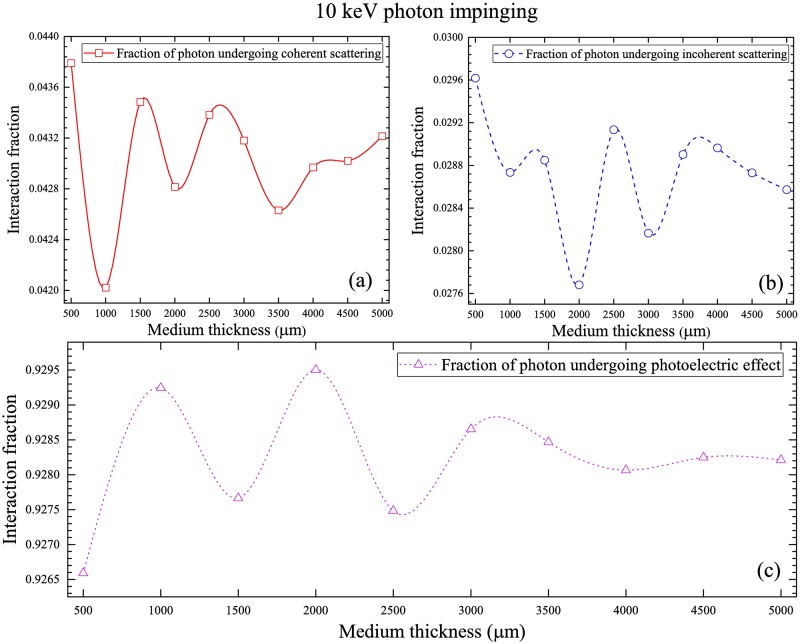
Fractions of interaction mechanisms of 10 keV photons for (a) coherent scattering, (b) incoherent scattering and (c) photoelectric interaction.

The fractions of interaction mechanisms of 10 keV photons are shown in Fig 8, which shows the order of decrease: photoelectric interaction > coherent scattering > incoherent scattering, which is controlled by the energy-dependent interaction cross-sections (Fig 3).

**Fig 9:**
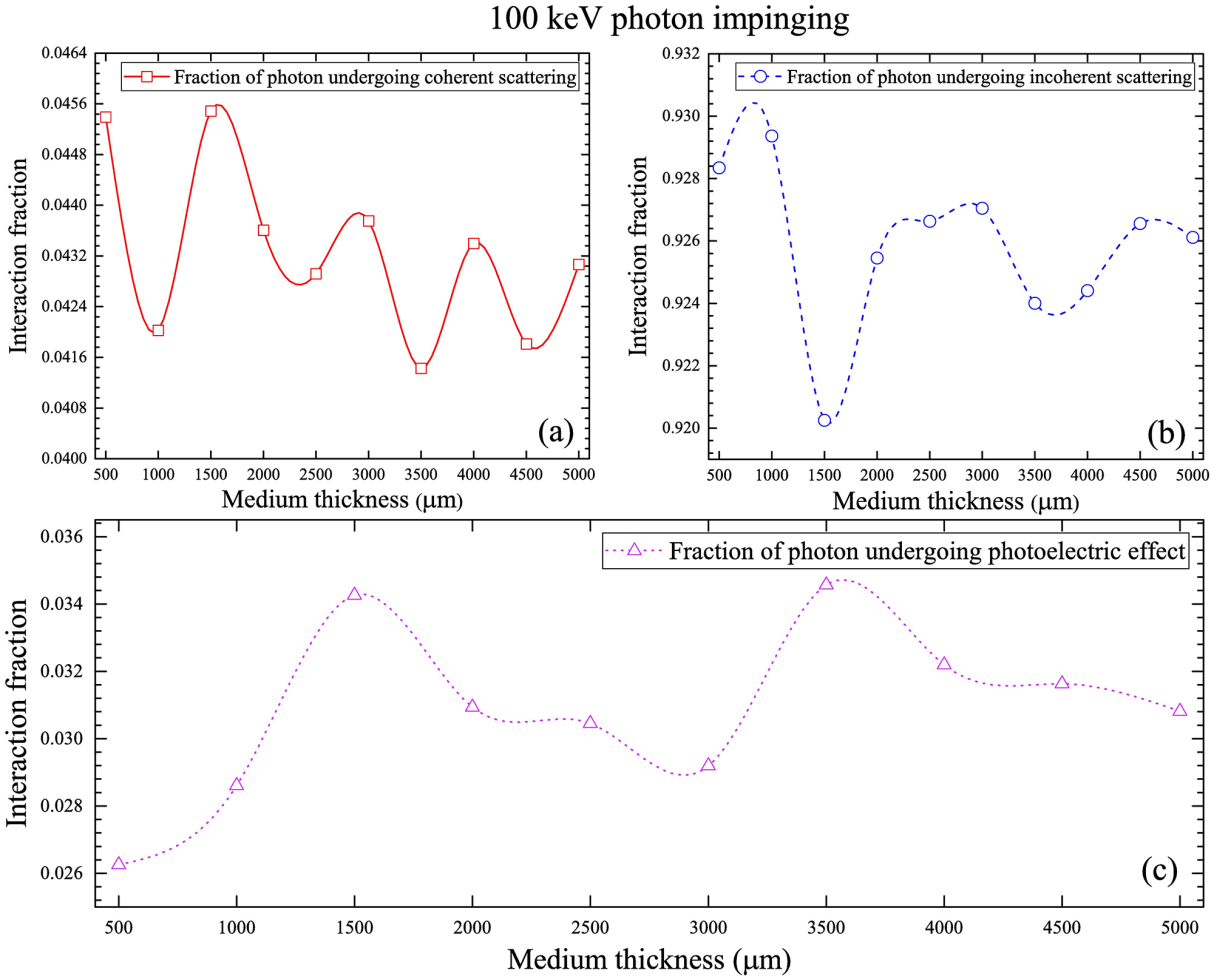
Fractions of interaction mechanisms of 100 keV photons for (a) coherent scattering, (b) incoherent scattering and (c) photoelectric interaction.

The fractions of interaction mechanisms of 100 keV photons are shown in Fig 9, which shows the order of decrease: incoherent scattering > coherent scattering > photoelectric interaction, which is again controlled by the energy-dependent interaction cross-sections (Fig 3).

**Fig 10:**
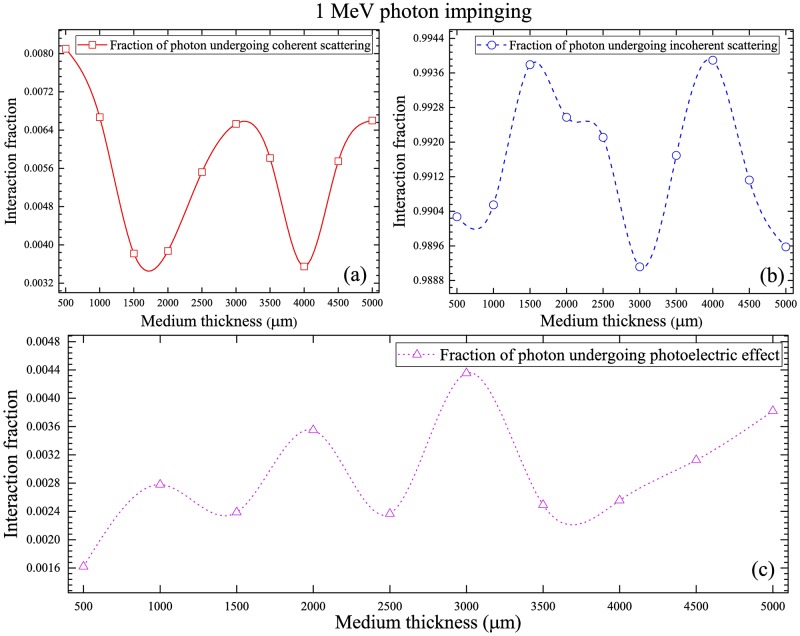
Fractions of interaction mechanisms of 1 MeV photons for (a) coherent scattering, (b) incoherent scattering and (c) photoelectric interaction.

The fractions of interaction mechanisms of 1 MeV photons are shown in Fig 10, which show the order of decrease: incoherent scattering > coherent scattering > photoelectric interaction, which is again controlled by the energy-dependent interaction cross-sections (Fig 3). The fractions of incoherent scattering increased noticeably when compared to 100 keV photons (see Figs 9(b) and 10(b)), which was due to the increased incoherent scattering cross-section at 1 MeV photon energy (see Fig 3). The corresponding reductions in the fractions of coherent scattering and photoelectric interaction were also observed.

### Electron ejection fraction during photoelectric interaction

**Fig 11:**
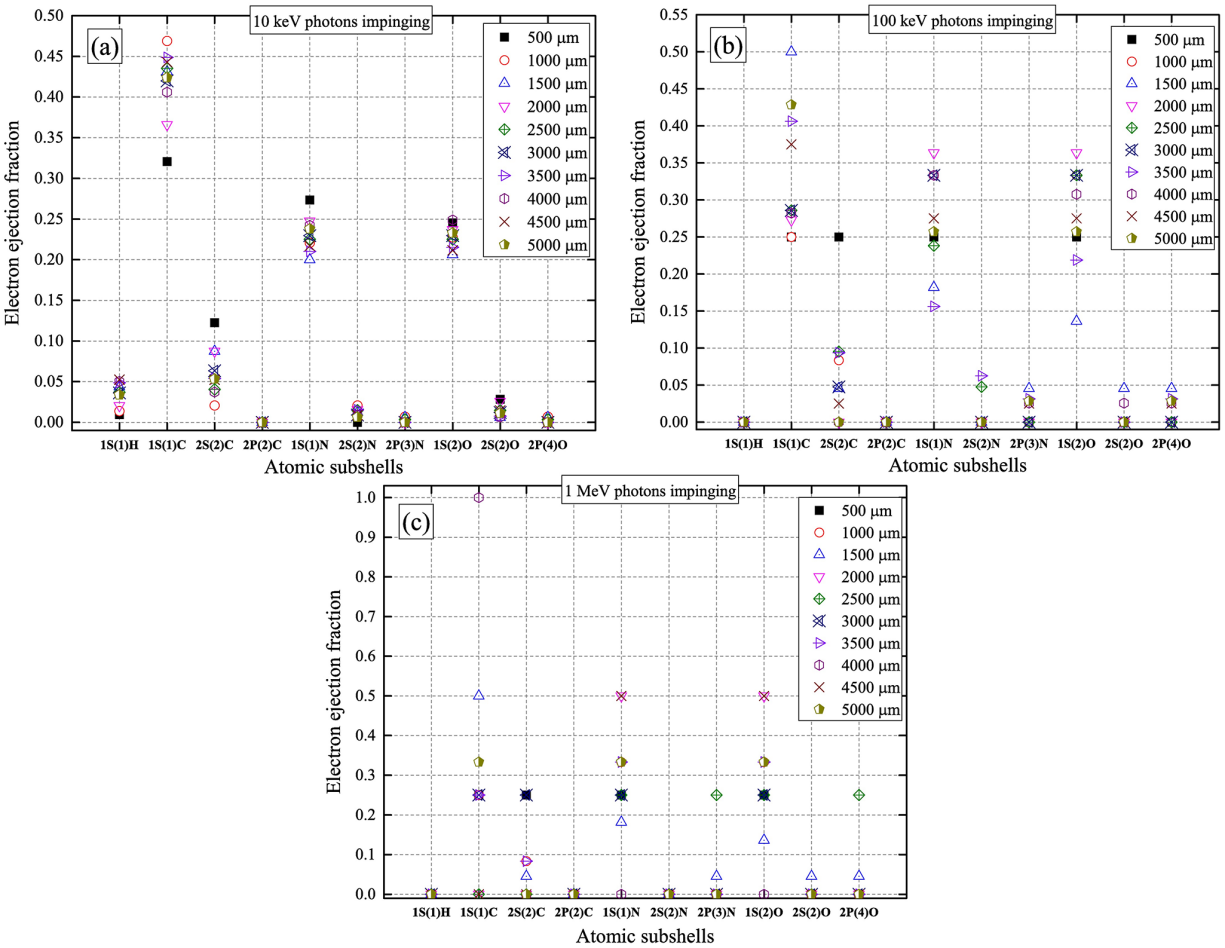
Electron ejection fractions during a photoelectric interaction from various atomic subshells for (a) 10 keV, (b) 100 keV and (c) 1 MeV incident photon energies, for a range of water medium thicknesses.

From the fractions of interaction mechanisms shown above, photoelectric interaction dominated for low-energy (10 keV) incident photons. For higher incident photon energies (100 keV and 1 MeV), the efficiency of photoelectric interaction significantly decreased but was not zero. Upon a photoelectric interaction, an electron was ejected. The electron ejection fractions from various atomic subshells for a range of water medium thicknesses for different incident photon energies are shown in Fig 11. In general, the 1s subshell had the highest electron ejection fractions. The variations for different nuclei were due to their different photoelectric interaction cross-sections (Fig 3). In particular, ^1^H had the lowest photoelectric interaction cross-sections, which were significantly reduced for higher incident photon energies (Fig 3a). In summary, the main factors which determined the electron ejection fractions were the incoming photon energy and water medium thickness. The nature of the ejected electrons was important when one needed to track the created secondary electrons from photoelectric interactions.

### Penetration fraction of photons

**Fig 12:**
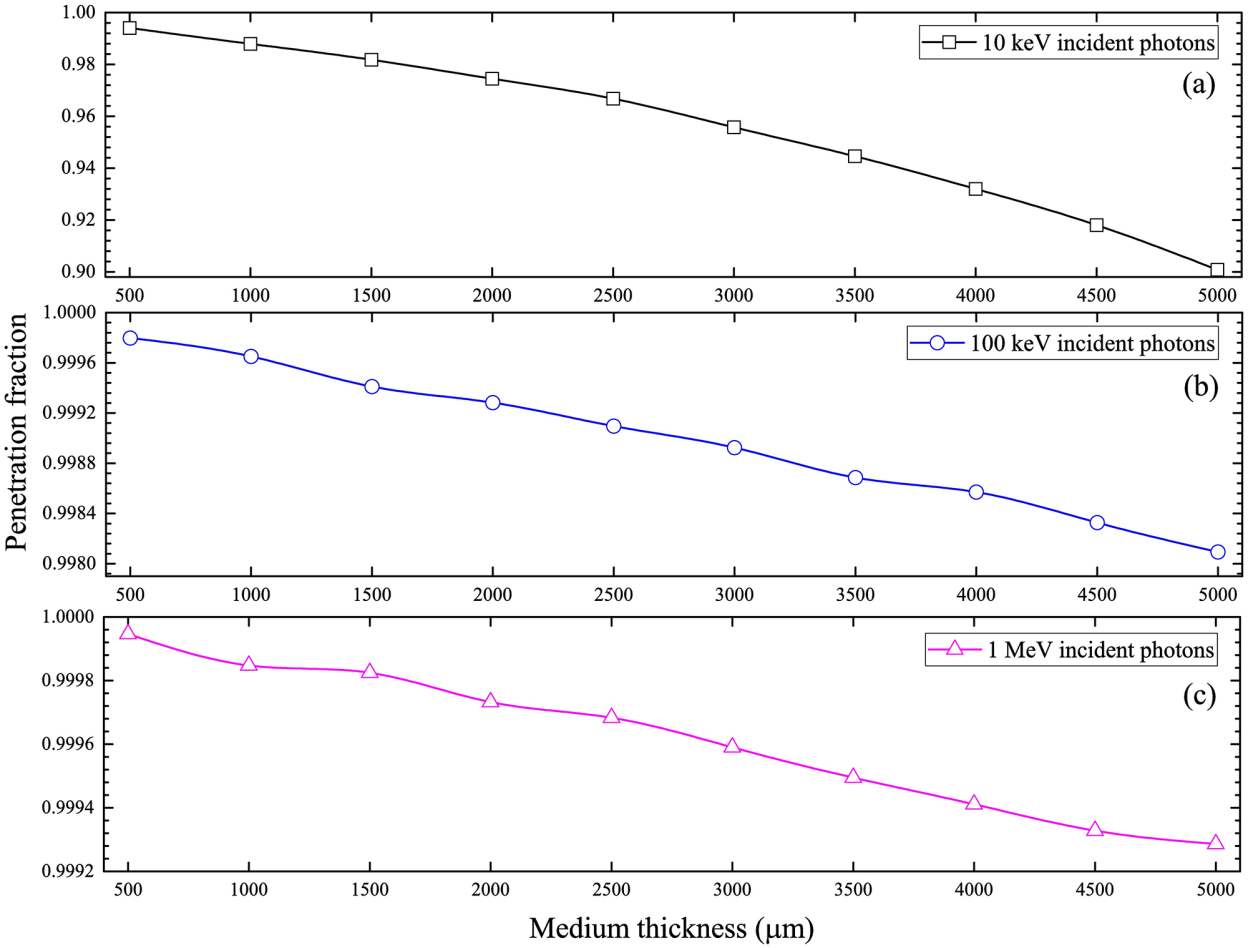
Penetration fractions of photons from the cell-medium system for photons with incident energies of (a) 10 keV, (b) 100 keV and (c) 1 MeV.

The penetration fractions of photons with incident energies of 10 keV, 100 keV and 1 MeV are presented in Fig 12. The penetration fractions were reduced for larger medium thicknesses, which was explained by the presence of more atoms in the medium leading to larger probabilities of photon interactions. Similar trends were also observed in our previous works [15,16]. On the other hand, the penetration fractions were enhanced for larger incident photon energies, which was due to the reduced interaction cross-sections.

### Angular distribution of photons exiting medium layer and subsequently undergoing interactions within cell layer

**Fig 13:**
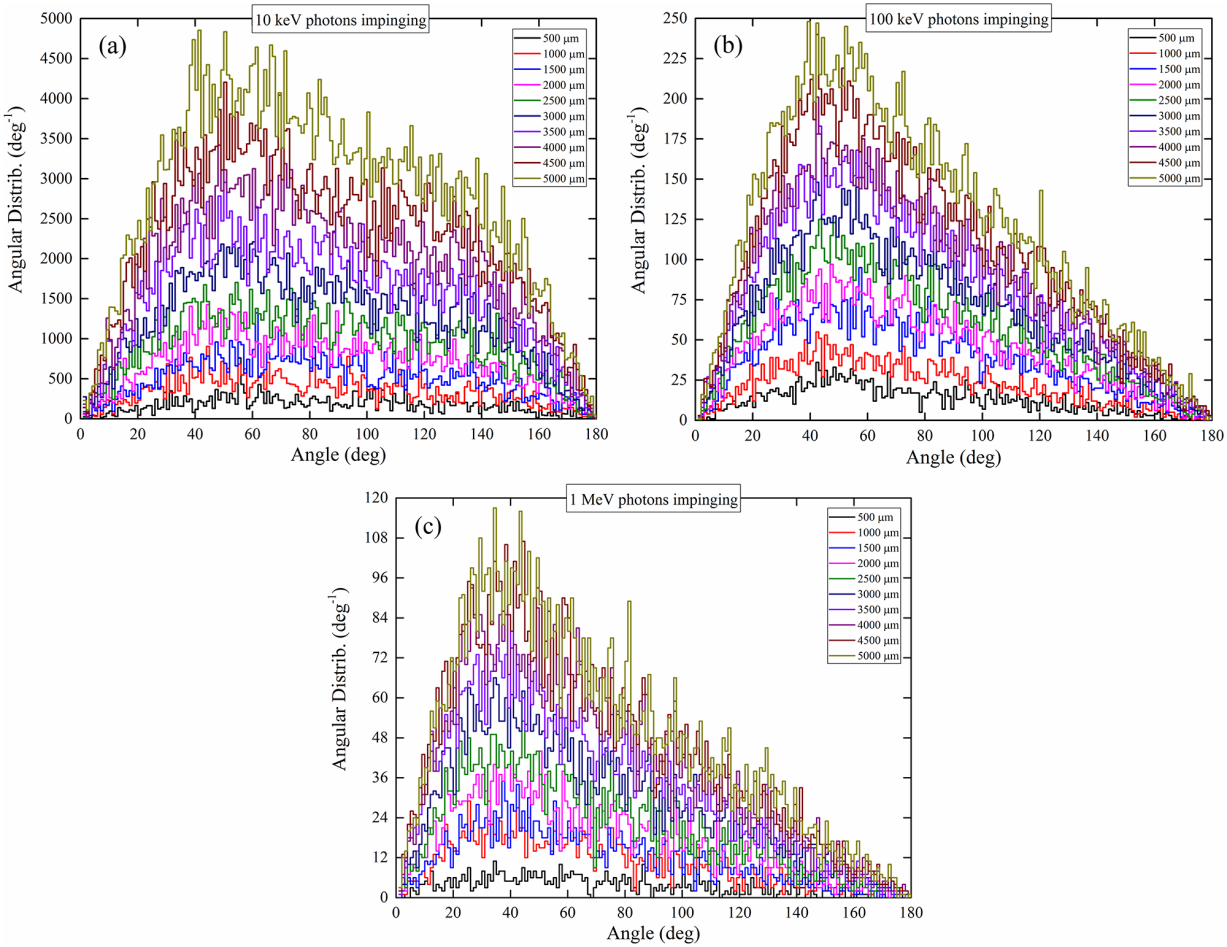
Angular distributions of photons exiting the medium layer and subsequently undergoing interactions within the cell layer, for incident photon energies of (a) 10 keV, (b) 100 keV and (c) 1 MeV.

The angular distributions (*dp*/*dθ*) of photons exiting the medium layer and subsequently undergoing interactions within the cell layer (hereafter abridged as the angular distributions) are presented in Fig 13. It is remarked that those photons which exited the medium layer but then traversed the cell layer without undergoing interactions will not be included in these distributions. Coherent scattering and incoherent scattering dominated for lower-energy (10 keV) and higher-energy (100 keV and 1 MeV) incident photons, respectively, and thus governed the angular distributions of photons exiting the medium layer and subsequently undergoing interactions within the cell layer for the corresponding incident photon energies.

**Fig 14:**
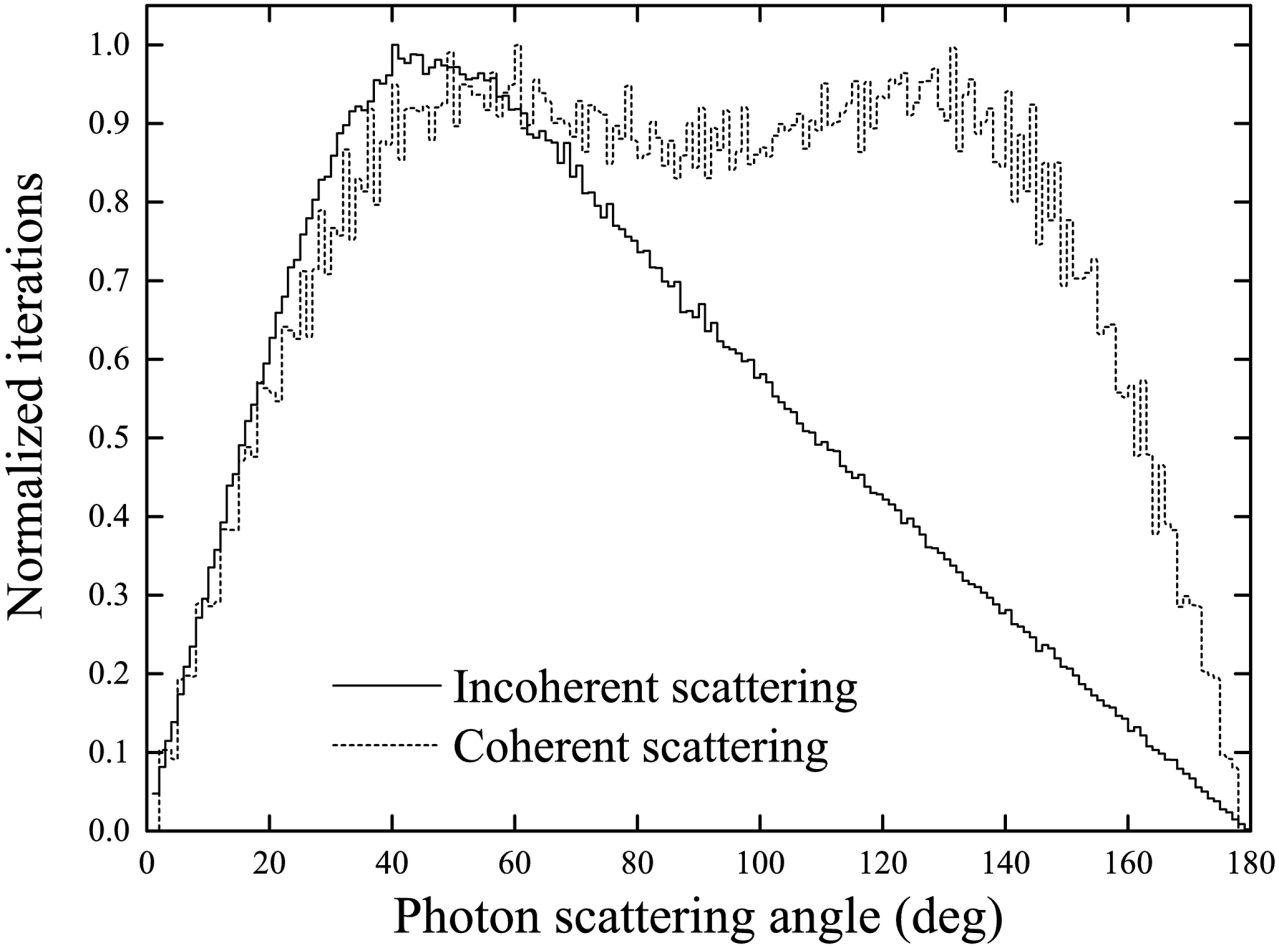
Cumulative distribution functions (CDFs) for incoherent and coherent scattering.

Incoherent and coherent CDFs are shown in Fig 14, which represent ideal angular distributions when either incoherent or coherent scattering events are scored. For photons perpendicularly impinging the surface of the medium layer, the angular distributions for photons with incident energies of 10 keV, 100 keV and 1 MeV were located at ~50°, ~45° and ~40°, respectively. For photons with large incident energy of 1 MeV, incoherent scattering was the dominating interaction mechanism (see Fig 10), so the angular distributions for these photons were close to the distributions for incoherent scattering and peaked at ~40°. For photons with lower incident energies, coherent scattering became more important, which shifted the peaks of the angular distributions for photons towards larger scattering angles (see Fig 14).

**Fig 15:**
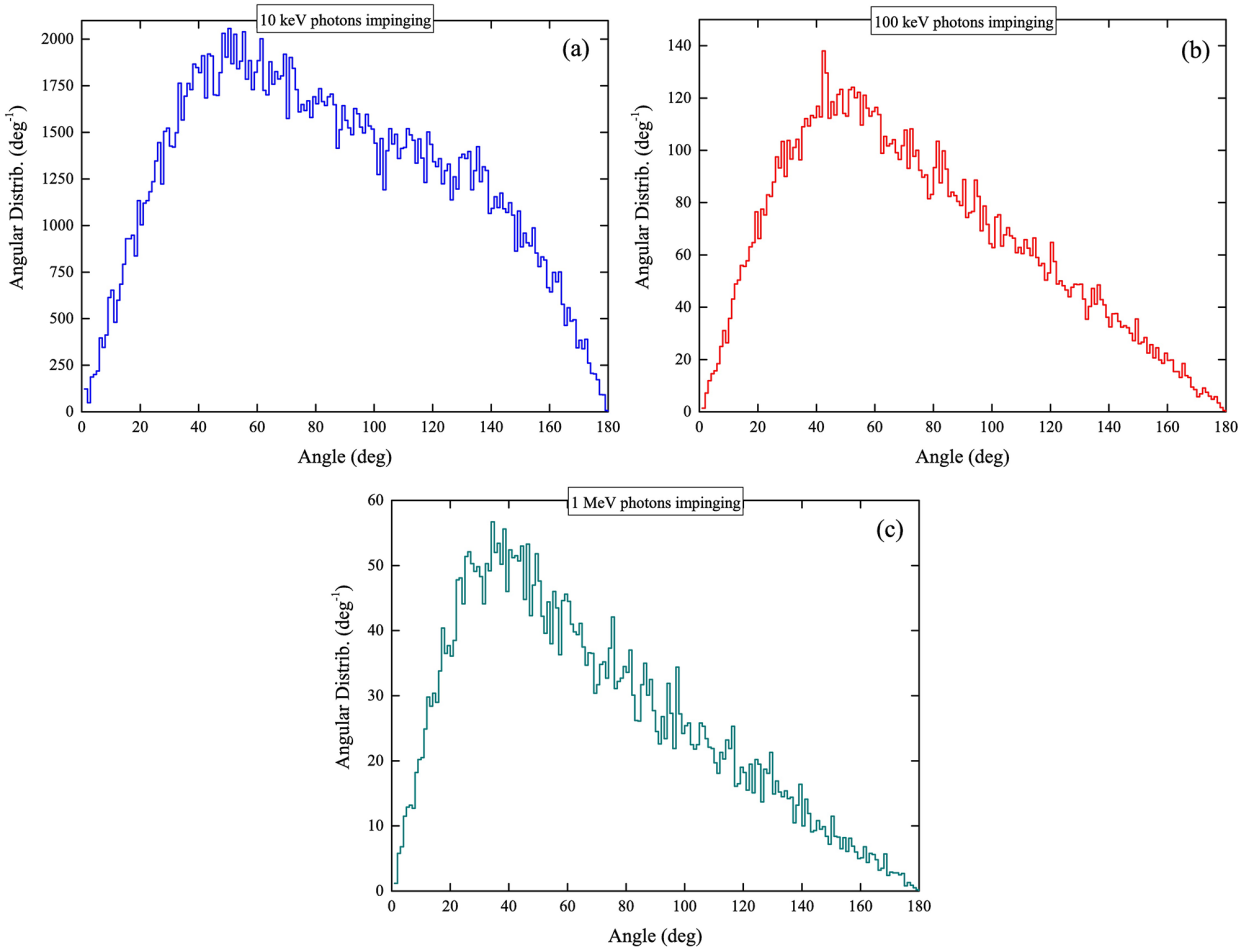
Average angular distributions of photons exiting the medium layer and subsequently undergoing interactions within the cell layer, for incident photon energies of (a) 10 keV, (b) 100 keV and (c) 1 MeV.

The areas under the curves for the angular distributions in Fig 13, i.e., ($\int_{\theta_{min}}^{\theta_{max}}\frac{dp}{d\theta }d\theta$), represented the integrated numbers of photons (referred to as “interacting photons”) that had exited the medium layer and subsequently underwent interactions within the cell layer [15]. From Fig 13, it was noted that the areas under the curves increased with the water medium thickness. In the presence of a thicker water medium layer, the incident photons lost larger fractions of their energies in the medium, so the photons exiting the medium had lower energies and thus had larger probabilities undergoing interactions within the cell layer (and therefore more interacting photons). Interestingly, the integrated numbers of “interacting photons” in the cell layer (areas under the angular distributions) were smaller for larger incident photon energies. To provide a clearer assessment, the average angular distributions (that were computed for different medium thicknesses) were obtained for specific incident photon energies, which are shown in Fig 15. Considering a fixed number of photons which entered the medium layer, the number of photons which exited the medium layer and subsequently underwent interactions within the cell layer for incident photon energy of 10 keV was ~19.4 times larger than the corresponding number for incident photon energy of 100 keV, which was in turn ~2.5 times larger than the corresponding number for incident photon energy of 1 MeV. The significant differences among the numbers of interacting photons were attributed to the increase in the interaction cross-section with the incident photon energy.

### Number of electron hits within a cell array and its dependence on administered dose

**Fig 16:**
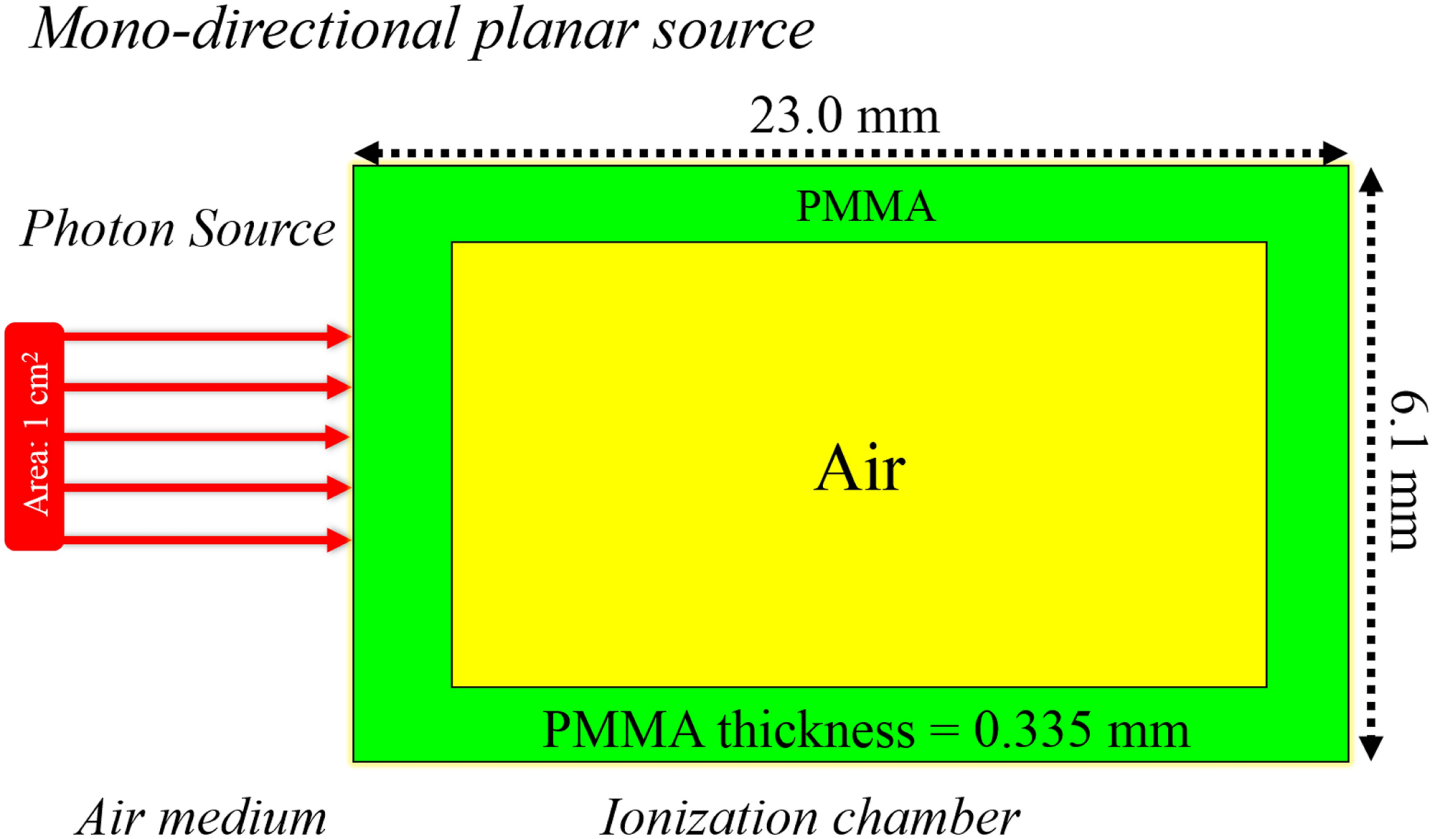
Schematic diagram showing photons impinging an ionization chamber (model TN 30 013, PTW Farmer^®^ Chamber).

In the present work, we studied a range of administered doses (*D*_*admin*_) from the low-dose range to the high-dose range commonly used by radiobiologists, namely, (1) 10 mGy, (2) 20 mGy, (3) 50 mGy, (4) 100 mGy and (5) 500 mGy. Usually, administered doses above and below 100 mGy are classified as high and low, respectively. Moreover, the usual practice is to monitor *D*_*admin*_ using a separate external radiation detector (e.g., ionization chamber). For illustration purposes in the present work, we simulated the doses recorded by a thimble ionization chamber (IOC) (model TN 30 013, waterproof PTW Farmer^®^ Chamber) as schematically shown in Fig 16, which was modelled in our previous work [25]. A source area of 1×1 cm^2^ was employed to determine the dose per photon from the source in the sensitive air volume of the IOC (*D*_*IOC*_) using the MCNP5 code. Four incident photon energies of 10 keV, 50 keV, 100 keV and 1 MeV were studied, and the doses delivered per incident photon in the sensitive volume of the IOC are shown in Table 2.

**Table 2:** Dose delivered per incident photon in the IOC from photons with different incident energies from the source.

Energy	10 keV	50 keV	100 keV	1 MeV
**Dose (Gy/photon)**	3.637×10^−12^	2.750×10^−13^	3.524×10^−13^	4.438×10^−12^

**Fig 17:**
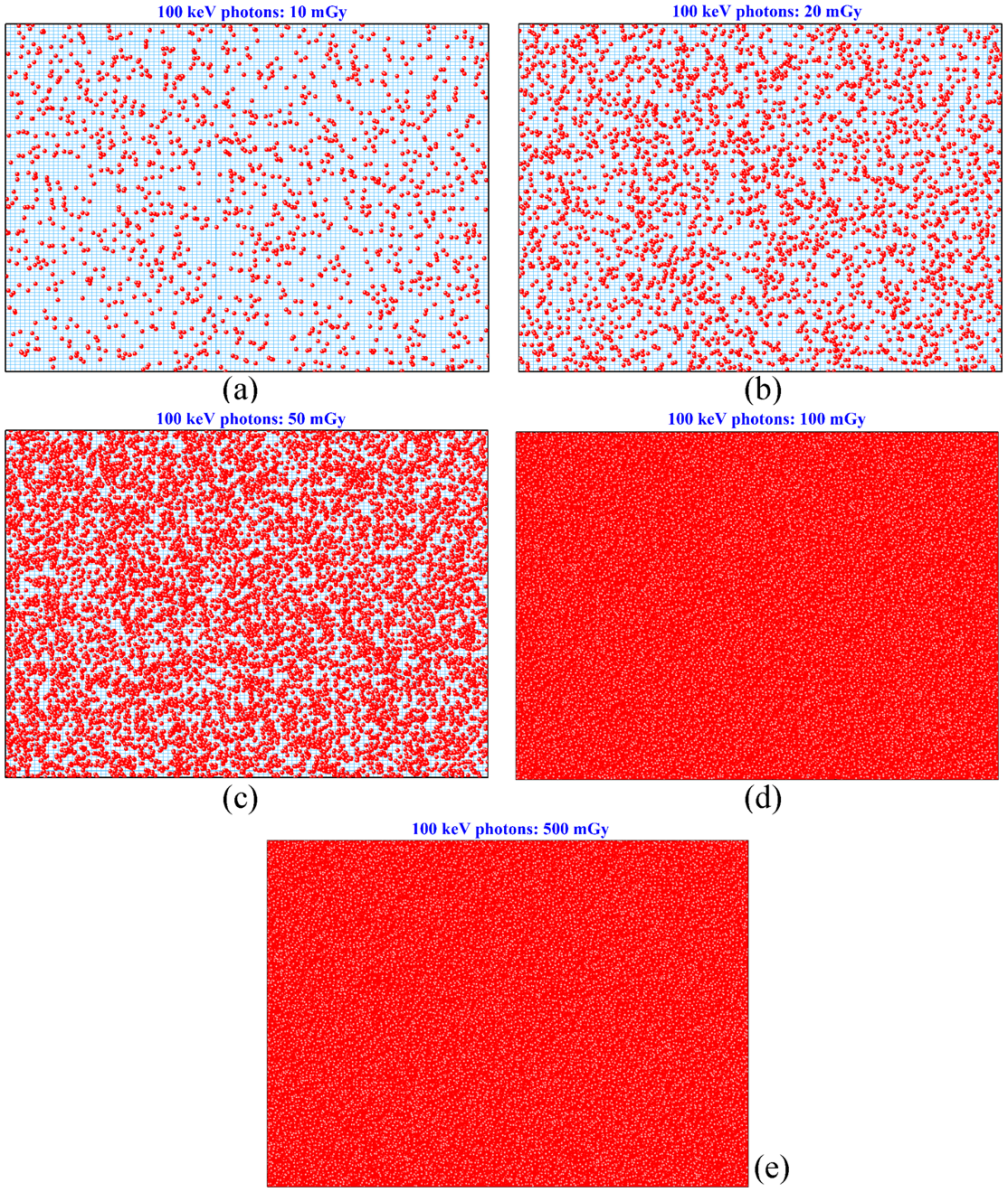
Electron hits on the exposed cell array with 10^4^ cells for 100 keV incident photons for administered doses of (a) 10 mGy, (b) 20 mGy, (c) 50 mGy, (d) 100 mGy and (e) 500 mGy.

From the results in Table 2, the total number of photons (*n*) emitted for a specific administered dose could be determined as *n = D*_*admin*_*/D*_*IOC*_, which was needed by the NRUphoton code. In the present work, pair production was not considered, and electrons were set in motion through incoherent scattering and photoelectric interaction. For the investigation here, only local hits were taken into account, i.e., the electrons were not transported, so the combined number of incoherent scattering and photoelectric interaction was referred to as “electron hits”. In the NRUphoton code, a cell layer with an area of 1×1 cm^2^ was exposed (with the understanding that it was possible that not all cells were hit by directly ionizing radiations, i.e., not all cells were irradiated), but a section of the exposed cell array consisting of 10^4^ cells was employed for convenient presentation of “electron hits”. For illustration purposes, the cells were assumed to be cuboids with a surface area of 10×10 μm^2^ and a thickness of 15 μm, and the water medium thickness was set to be 2600 μm (cf. Ref. [26]). The location of each electron hit was determined and plotted on the displayed cell array section. Sample results for 100 keV incident photons are shown in Fig 17 for five different administered doses.

The numbers of cells suffering at least one electron hit are summarized in Table 3 for different incident photon energies and for different administered doses. As expected, the factor of increase in the number of cells suffering at least one electron hit was similar to the factor of increase in the administered dose *D*_*admin*_, until all cells had suffered at least one electron hit. For larger incident photon energies, the numbers of cells suffering at least one electron hit became smaller, which was attributed to the reduction in the photon interaction cross-section. From the results shown in Figs 8 to 10, photoelectric interaction and incoherent scattering dominated for lower-energy (10 keV) and higher-energy (100 keV and 1 MeV) incident photons, respectively. It is remarked that the photoelectric interaction cross-sections were significantly larger than the incoherent scattering cross-sections (see Fig 3). The threshold administered dose at which all cells in the array suffered at least one electron hit increased with the incident photon energy. At higher incident photon energies, a larger number of incident photons (i.e., a higher *D*_*admin*_) would be required to compensate for the reduction in the total interaction cross-section (Fig 3). These results highlighted the importance of the administered dose in radiobiological experiments, since the radiobiological effects critically depended on the percentage of irradiated cells in the studied cell population. In particular, the threshold administered doses at which all cells in the array suffered at least one electron hit might provide hints on explaining the intriguing observation that radiation-induced cancers can be statistically detected only above the threshold value of ~100 mSv, and thus on reconciling controversies over the linear no-threshold model.

**Table 3:** Number of cells suffering at least one electron hit for different incident photon energies and for different administered doses.

Energy/D_admin_	10 mGy	20 mGy	50 mGy	100 mGy	500 mGy
**10 keV**	9771	10000	10000	10000	10000
**50 keV**	4203	8394	10000	10000	10000
**100 keV**	1334	2664	6114	10000	10000
**1 MeV**	196	393	985	1961	9848

## Conclusions

The mechanisms underlying photon interactions in radiobiological experiments were studied in detail using our developed NRUphoton computer code. The present code was benchmarked against the MCNP5 code by comparing the photon dose delivered to the cell layer below the water medium. A number of conclusions were made:

1. The interaction fractions of photons with ^16^O nuclei were the largest compared to other nuclei mainly due to the significantly larger interaction cross-sections of ^16^O nuclei. The interaction fractions decreased in the following order: ^16^O > ^12^C > ^14^N > ^1^H. Bulges in the interaction fractions (versus water medium thickness) were observed, which reflected changes in energies of the propagating photons as a result of the water medium thickness as well as changes in the energy-dependent photon interaction cross-sections.
2. Photoelectric interaction and incoherent scattering dominated for lower-energy (10 keV) and higher-energy (100 keV and 1 MeV) incident photons.
3. The fractions of electron ejection from different nuclei were mainly governed by the photoelectric effect cross-sections, and the fractions from the 1s subshell were the largest.
4. In general, the penetration fractions decreased with increasing medium thickness, and increased with increasing incident photon energy, the latter being explained by the corresponding reduction in interaction cross-sections.
5. The areas under the angular distribution curves of photons exiting the medium layer and then subsequently undergoing interactions within the cell layer became smaller for larger incident photon energies.
6. The number of cells suffering at least one electron hit increased with the administered dose. For larger incident photon energies, the numbers of cells suffering at least one electron hit became smaller, which was attributed to the reduction in the photon interaction cross-section.

In Monte Carlo simulation studies based on the cross-sectional data used for water medium and cell layers, uniform irradiation of all cells was assumed, and normal incidence of photons onto the medium layer was assumed to be perpendicular. However, in actual ionization scenarios, the cells might not be irradiated uniformly, e.g., when the cells were not 100% confluent and homogeneously distributed in the irradiation dish, or when the irradiation beam size could not cover all cells. Moreover, the photons might not have normal incidence onto the medium layer, e.g., when conical beams were used. These might lead to some discrepancies between the simulated results and the actual scenarios, although the discrepancies were expected to be small.
